# Sutureless Aortic Prosthesis Implantation: the First Brazilian Experience with Perceval Device

**DOI:** 10.5935/1678-9741.20160065

**Published:** 2016

**Authors:** Ana Paula Tagliari, Leandro de Moura, Luiz Henrique Dussin, Eduardo Keller Saadi

**Affiliations:** 1 Clinics Hospital of Porto Alegre (HCPA), Federal University of Rio Grande do Sul (UFRGS), Porto Alegre, RS, Brazil.

**Keywords:** Cardiovascular Surgical Procedures, Aortic Valve Stenosis, Heart Valve Prosthesis Implantation

## Abstract

This is a report of the first Brazilian experience with the Perceval sutureless aortic prosthesis in two patients with severe aortic stenosis. Transesophageal echocardiography was used during the procedure. The aortotomy was performed 1 cm above the sinotubular junction, followed by leaflets removal and decalcification. Correct valve size was selected, device released and an accommodation balloon used. The cardiopulmonary bypass times were 47 and 38 min and the cross-clamp times were 38 and 30 min. There was a significant decrease in mean gradients (41 and 75 mmHg preoperatively; 7 and 8 mmHg postoperatively). There was no major complication or paravalvular leak.

**Table:** 

Abbreviations, acronyms & symbols	
EuroSCORE	=Mean logistic European System for Cardiac Operative Risk Evaluation
NYHA	=New York Heart Association
STS	=Society of Thoracic Surgeons
TAVI	=Transcatheter aortic valve implantation

## INTRODUCTION

Degenerative aortic stenosis affects approximately 3% of the population aged 75 years and over and it's the most common heart valve disease. The mean survival of angina or heart failure symptoms development is two years^[1]^.

The aortic valve replacement significantly improves survival and it has been still considered the treatment of choice. However, one third of the patients are considered not to be candidates for standard surgical approaches, either for anatomical abnormalities, previous thoracic surgery or chest radiation, comorbidities or overall frailty^[2]^.

In this scenario, transcatheter aortic valve implantation (TAVI) has assumed an important role. It was initially designed for high risk patients, but now it can be used even in moderate risk ones [Society of Thoracic Surgeons (STS) score from 4% to 8%]^[3]^. Nevertheless, not all patients are candidates for TAVI, some due aortic root or valve abnormalities, others because additional cardiac procedures are needed (other valve replacement, coronary artery bypass grafting or repair of the aortic root).

As alternatives to these difficulties, modern sutureless aortic prostheses have emerged. Since now, the Perceval prosthesis (LivaNova Biomedica Cardio Srl, Sallugia, Italy) has been considered the device that surgeons have more expertise. Its surgical implant allows complete and safe annulus decalcification and can be performed through minimally invasive procedures.

A special subgroup of patients who could benefit from this device is that with a very small annulus, that could require aortic annular enlargement during aortic valve replacement or the elderly patients with comorbidities and calcified aorta.

Considering that the Perceval sutureless aortic prosthesis is the most worldwide studied and implanted valve, this report aims to publish the first Brazilian experience with this device implantation.

## METHODS

A 73 years-old man was the first patient who underwent surgery. He had been diagnosed with severe aortic stenosis one year ago and nowadays was presenting dyspnea New York Heart Association (NYHA) class I-II. His preoperative echocardiography showed parameters consistent with severe aortic stenosis (peak and mean gradient 81/41 mmHg, respectively; peak velocity 4.5 m/s and valve area 0.7 cm^2^).

The second case was a 63 year-old man diagnosed with aortic stenosis two years ago and now presenting dyspnea NYHA class II. His echocardiography were similar to the previous patient (peak and mean gradient 129/75 mmHg, respectively; peak velocity 4.5,m/s and valve area 0.9 cm^2^).

The mean logistic European System for Cardiac Operative Risk Evaluation (EuroSCORE) and STS score of these patients were 0.81% and 0.82%; 0.99% and 0.97%, respectively and their body surface area were 1.99 and 1.71 m^2^.

The surgery was performed in May 2016 at the Hospital de Clínicas de Porto Alegre, Brazil, according to Dedeilias et al.^[4]^ technique description. After a median full sternotomy, a routine cannulation to the cardiopulmonary bypass was performed with aortic cannula at the distal part of the ascending aorta and a two-stage venous cannula at the right atrium. The cross clamp was applied as distal as possible in the ascending aorta and a transverse aortotomy was performed 1 cm distal to the sinotubular junction (approximately 3.5 cm above the valve ring), followed by direct cannulation of the coronary ostia and delivering of cold cardioplegia solution. After the calcified native aortic valve was removed and the aortic annulus decalcified and measured, 3 guiding sutures were positioned 2 mm below the nadir of the native leaflet insertion line of each valve sinus. These sutures were passed through the corresponding eyelets in the prosthetic inflow ring, as a reference for alignment of the inflow section of the prosthesis with the insertion plane of the native leaflets.

**Fig. 1:**
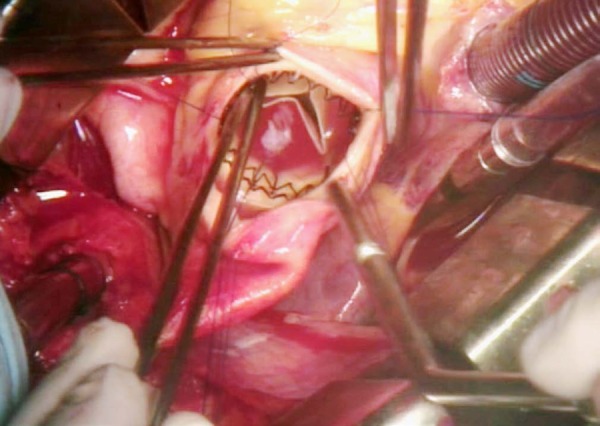
Perceval sutureless aortic prosthesis deployed.

The prosthesis valve was released into two phases: first, the inflow section of the valve, followed by the opening of the outflow part, then a post-implant dilatation was performed with a balloon catheter at pressures of 4 atm for 30 seconds (Figure 1). Once the prosthesis was completely deployed, the guiding sutures were removed.

Three-dimensional transesophageal echocardiography performed by experienced echocardiographer was available during all the procedures.

In order to allow expansion and anchorage of the device and reduce migration risk, the ratio between the sinotubular junction diameter and the aortic valve annulus couldn't exceed 1.3 - prerequisite obtained in both cases (1.0 e 1.2).

## RESULTS

The procedures were performed with participation of a proctor of the University of Graz in Austria. The cardiopulmonary bypass times were 47 and 38 minutes and the cross-clamp times were 38 and 30 minutes. Mechanical ventilation weaning, postoperative bleeding control and intensive care unit stay were performed according to the routine of the cardiovascular surgery postoperative care.

Postoperative echocardiography showed mean gradients of the 7 and 8 mmHg and there was no paravalvular leak.

Both patients had favorable postoperative clinical course. None of them had any major complication nor atrioventricular block determining need of pacemaker implant. They were discharged on the tenth day after surgery.

## DISCUSSION

The constant search for less invasive surgery techniques is extremely relevant for modern cardiovascular surgery. Once the aortic stenosis is the most frequent valvular disease, keeping pace with advances in its surgical treatment maintains a surgery group competitive and provides an opportunity for patients to have access to the best treatments available.

**Fig. 2:**
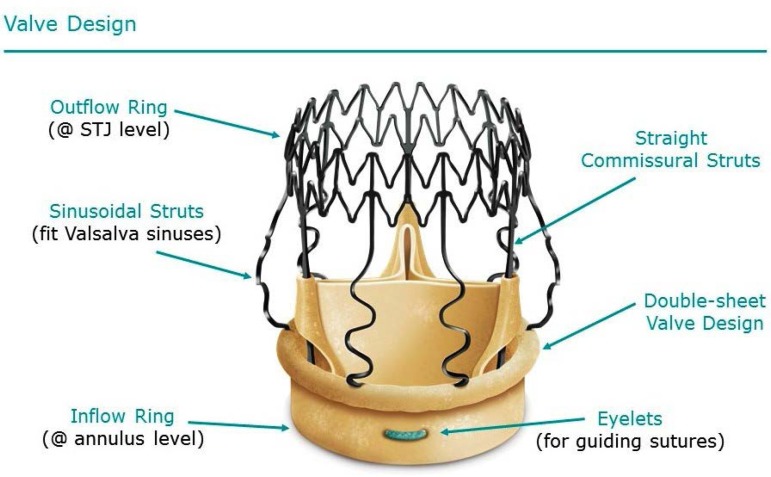
Perceval structure (courtesy LivaNova).

Even though worldwide several prospective studies and systematic reviews have demonstrated safety and excellent hemodynamic results with the Perceval sutureless bioprosthesis, this device had not been used in Brazil yet. It's a self-expanding bovine bioprosthesis valve mounted in a Nitinol stent and designed to preserve aortic sinuses and sinotubular junction (Figure 2).

When we compare the aortic valve replacement using sutureless prosthesis with the conventional technique, we realize not only aortic cross-clamp, cardiopulmonary bypass and surgery times significantly shorter, but also less blood transfusion required, lower intubation time, shorter intensive care unit stay, hospital stay and lower incidence of postoperative atrial fibrillation and respiratory insufficiency. This lower rate of postoperative complications resulted in reduced resource consumption in the sutureless group with a total cost saving of approximately 25%^[5]^.

It is worth mentioning that Brazil already has experimental studies in animal models in order to develop a similar device (Inovare Alpha - Braile Biomédica, São José do Rio Preto, SP, Brazil). The results were published in 2015 by Gomes et al.^[6]^ and demonstrated excellent clamp time (mean of 7 min, ranging from 6 to 10 minutes) and no paravalvular leak after the prosthesis implant. It is now ongoing in Brazil a trial comparing Alpha with conventional aortic valve replacement in patients with combined procedures. There is another sutureless valve (Intuity – Edwards Lifesciences Corporation, Irvine, CA, USA) that has just been approved some months ago for clinical use in Brazil. This device needs 3 sutures to be tight at the nadir of each cusp.

## CONCLUSION

We reported the first national experience with the Perceval sutureless aortic prosthesis. This device proved safety and excellent hemodynamic results. In our two cases we observed a reduction of mean gradients higher than would be expected from conventional valve replacement.

**Table:** 

Authors’ roles & responsibilities	
APT	Conception and design study; realization of operations and/or trials; manuscript writing or critical review of its content; final manuscript approval
LM	Realization of operations and/or trials; final manuscript approval
LHD	Realization of operations and/or trials; final manuscript approval
EKS	Conception and design study; realization of operations and/or trials; manuscript writing or critical review of its content; final manuscript approval
